# Toward Designs of Workplace Stress Management Mobile Apps for Frontline Health Workers During the COVID-19 Pandemic and Beyond: Mixed Methods Qualitative Study

**DOI:** 10.2196/30640

**Published:** 2022-01-13

**Authors:** Beenish Moalla Chaudhry, Ashraful Islam, Monica Matthieu

**Affiliations:** 1 University of Louisiana at Lafayette School of Computing and Informatics Lafayette, LA United States; 2 Saint Louis University College for Public Health and Social Justice, School of Social Work Saint Louis, MO United States

**Keywords:** mental health, stress, mHealth, frontline health worker, design requirements, pandemic, COVID-19, design, intervention, burnout, perspective, need, user design

## Abstract

**Background:**

In recent years, mobile apps have been developed to prevent burnout, promote anxiety management, and provide health education to workers in various workplace settings. However, there remains a paucity of such apps for frontline health workers (FHWs), even though FHWs are the most susceptible to stress due to the nature of their jobs.

**Objective:**

The goal of this study was to provide suggestions for designing stress management apps to address workplace stressors of FHWs based on the understanding of their needs from FHWs’ own perspectives and theories of stress.

**Methods:**

A mixed methods qualitative study was conducted. Using a variety of search strings, we first collected 41 relevant web-based news articles published between December 2019 and May 2020 through the Google search engine. We then conducted a cross-sectional survey with 20 FHWs. Two researchers independently conducted qualitative analysis of all the collected data using a deductive followed by an inductive approach.

**Results:**

Prevailing uncertainty and fear of contracting the infection was causing stress among FHWs. Moral injury associated with seeing patients die from lack of care and lack of experience in handling various circumstances were other sources of stress. FHWs mentioned 4 coping strategies. Quick coping strategies such as walking away from stressful situations, entertainment, and exercise were the most common ways to mitigate the impact of stress at work. Peer support and counseling services were other popular methods. Building resilience and driving oneself forward using internal motivation were also meaningful ways of overcoming stressful situations. Time constraints and limited management support prevented FHWs from engaging in stress management activities.

**Conclusions:**

Our study identified stressors, coping strategies, and challenges with applying coping strategies that can guide the design of stress management apps for FHWs. Given that the pandemic is ongoing and health care crises continue, FHWs remain a vulnerable population in need of attention.

## Introduction

Work stress is recognized as the response workers have when they are presented with work responsibilities that do not match their knowledge and abilities and that challenge their ability to cope [1]. Workers may appreciate some demands and pressures to stay alert, motivated, able to work, and learn, depending on their abilities and available resources. However, when these pressures and demands become exorbitant and persist for a long time, they manifest as stress, which can be damaging to worker’s health and workplace performance. During the COVID-19 pandemic, frontline health workers (FHWs) were tasked with delivering essential health services in circumstances that surpassed their knowledge and abilities, challenging their ability to cope. Several studies reported high rates of stress-related health issues such as clinically significant depression (23.2%), anxiety (22.8%), and insomnia (38.9%) among the FHWs in primary care and hospital settings [2,3]. Even though similar rates of mental health issues have been reported during and following previous viral outbreaks and pandemics (eg, (Ebola, severe acute respiratory syndrome, and Middle East respiratory syndrome) [4,5], proactive solutions for managing stress do not seem to exist for FHWs. The fear of stigma, time restrictions, and expectation of maintaining phlegm are the main reasons why the mental health of FHWs is often ignored [6,7]. Moreover, it is generally assumed that FHWs are well equipped to handle their mental health challenges by the virtue of their professional training. This understanding needs to change because without adequate support to manage work-related stress, FHWs are at risk of developing a wide range of health problems [2], and the society is at risk of losing a workforce responsible for delivering essential health services in times of crisis.

Stress management interventions come in several varieties. The interventions that directly target sources of stress to improve workers’ well-being are called primary interventions. They are concerned with modifying the content and context of the workplace, such as redesigning job descriptions, providing career development opportunities, providing flexible work schedules, encouraging goal-setting, and implementing team-building and diversity [8]. The secondary interventions aim to decrease the intensity or duration of stress response after its occurrence to minimize the damage. These interventions aim to increase workers’ resilience by teaching them specific strategies in physical (eg, meditation and relaxation exercises), emotional (eg, externalization of negative emotions), and cognitive (eg, cognitive-behavioral techniques) domains [8]. The tertiary interventions are about treatment and rehabilitation of individuals to facilitate return to work after the damage. They typically include options such as counseling support and medical interventions [9].

Technology can help deliver such interventions at the organizational or individual level [10]. The organization-level technological interventions are aimed at making organizational practices changes that either target all workers or a specific group of workers. The individual-level technological interventions are aimed at helping workers develop coping skills to manage and control their own stress responses in situations and circumstances they perceive as being stressful. Mobile apps are suitable for delivering both types of stress management interventions as they are easily customizable and can remove barriers to help-seeking and reach FHWs “where they are.” Research indicates that mobile apps can reinforce healthy habits and scaffold recovery processes via streamlined designs [11]. They can provide easy access to self-help reference materials while preserving privacy needs and time constraint issues of individuals [12]. Wearable devices, on the other hand, can also passively monitor behaviors to assess user’s needs and, in response, curate context-aware, personalized, adaptive, and anticipatory interventions [13]. In other words, mobile apps provide the flexibility to deliver just-in-time and suitable interventions in users’ context. Such interventions can help reduce barriers to decision-making and action taking for positive behavior changes [14-16].

During the COVID-19 pandemic, there was an increased interest in developing mobile apps to preserve the mental well-being of FHWs (Table 1). HeroesHealth [17], Be + against COVID [18], Wellness Hub [19], and Clinicovery [20] are among the apps that were developed during the pandemic to address the stress management needs of different types of health care workers. With these apps, FHWs can assess their own mental health by answering survey questions and reviewing status reports. Another important component included in these apps is professional support, especially information about free and low-cost mental health services. Although many incorporate evidence-based stress management content, the focus of existing project has been development and implementation from a technological perspective [21]. The involvement of the target users—that is, FHWs in the conceptualization and development of these tools—was either limited or missing. The evidence of the effectiveness of mobile apps for addressing stress and other mental health symptoms of FHWs who are involved in the current health crisis is also not available [18]. Moreover, existing stress management interventions do not use any theoretical understanding of stress to explain the mechanism of the intervention [22].

**Table 1:** Feature summary of COVID-19 mental health–related mobile apps.

App name	Features
Heroes Health [17]	Mental health tracking over time via periodic assessments. Links to instant support and mental health resources. Summaries of health workers’ data for organizational use. Communication between health workers and organizations.
Be + against COVID [18]	Rapid resources for combating COVID-19. Assessments to self-evaluate the ability to adjust to the crisis.
Wellness Hub [19]	Standardized mental health surveys. Score-based feedback consisting of resilience-building exercises, resource links, etc. Private digital journal for self-reflection. Relaxation videos and other resources.
Clinicovery [20]	Written and audiovisual content for addressing various mental health issues. Daily prompts (notifications) with brief questionnaires to monitor mental health status. Short messages offering tailored information and resources based on the participants´ responses.

To allay these gaps, we referenced stress, appraisal, and coping theories within the person-environment transactional framework [23,24] to investigate stressors and coping strategies among FHWs. The framework suggests that when an individual reacts to an event on the basis of the notion that it will be harmful to his/her personal well-being, the event becomes a psychological stressor. It also accounts for an individual’s coping skills and overall coping mechanisms that can provide directions for designing interventions. Using this theoretical framework as the basis, we explored stressors during pandemic and stress management strategies from FHWs’ perspectives. Based on our findings, we discuss implications for designing stress management mobile apps. We make two contributions: first, our human-centered approach elucidates the wants and needs of the target users, which has theoretical implications for conceptualizing the understanding of stress; second, our design requirements and suggestions may be useful to other researchers who are designing stress management mobile apps for FHWs.

## Methods

### Methods Overview

The goal of this study was to provide suggestions for designing mobile apps to address workplace stressors of FHWs on the basis of the Transactional Framework of Stress. Compared to the other approaches to understanding the mechanisms of stress, this framework provides practitioners with all the necessary constructs to work with stress victims by tapping into their existing and personal coping skills and strategies. It has also been previously used to understand implications for clinical practice in working with individuals experiencing acute stress following a disaster in community [25]. The framework recommends taking the person-centered approach; that is, it encourages the involvement of target users in all stages of intervention design, so their needs can be identified, and perspectives integrated [26]. To elicit user needs for the design of the stress management app, we used multiple methods to engage with the target users; that is, FHWs.

### Document Analysis

When we started this study in July 2020, the COVID-19 pandemic was at its peak in the United States, and it was challenging for us to reach the target users. We initiated contact with a few FHWs but found them overstretched with extra responsibilities owing to the pandemic and not interested in participating in the study. Consequently, we resorted to gathering interviews of FHWs, which had been published in reputable web-based news portals such as CNBC and CNN.

Using a combination of words such as “frontline health worker,” “stress,” “mental health,” and “covid-19,” we searched for relevant articles on various web-based news sites between December 2019 and October 2020 using the Google search engine. Over 200 articles were found, of which, we selected 41 that discussed challenges and experiences of health workers during the pandemic. More specifically, 3 researchers independently reviewed these articles to identify content relevant for this research. We selected the following content: (1) quotes from interviews with FHWs (ie, FHWs’ views and comments) and (2) quotes discussing workplace stressors and related concerns.

### FHW Survey

By October 2020, we had completed the analysis of the gathered interviews. By that time, the pandemic was also under relative control in several US states. To triangulate our findings from the analysis, we prepared a survey in Qualtrics software, informed by the transactional framework of stress. We obtained approval to conduct the survey from the ethical review board of our home institution. Participants were recruited via convenience sampling; the survey was open (publicly available), and emailed to several mailing lists consisting of various FHWs such as nurses and physician assistants. Participation in the survey was voluntary, and no initial contact about the survey with the target audience had been made. No compensation was provided. The main inclusion criteria included being >18 years old and working at a large health care facility. The exclusion criterion was working in small clinics or health centers. The survey remained open from October 4-24, 2020.

The purpose of the survey was to further our understanding of FHWs’ needs and challenges regarding stress during the pandemic by understanding their stressors and coping strategies. The first part of the survey was the informed consent process, where participants were briefed about the study purpose and informed about the investigator. In addition, participants were informed that the survey completion time was 30-45 minutes, and that the data will be stored for 1 year after the closing of the survey. The second part of the survey collected demographic information (eg, age, gender, years of experience, educational background, and job title) and asked the remaining questions listed in Table 2. No identifying information was collected. The survey questions were not randomized, and all main survey questions were on the same page (for a total of 3 survey screens, including one each for informed consent and demographic data). The IP information collected by the Qualtrics was used as a proxy to ensure each survey response was from a different individual.

**Table 2:** Survey questions (questions are categorized in accordance with the subsections describing associated findings).

Question #		Question text
**Specific aim 1: to identify the sources of stress or stressors during pandemic and normal situations**		
	1.1	What kinds of stressors do you face at work?
	1.2	What kinds of stressors did you face at work during the pandemic?
**Specific aim 2: to examine the stress management strategies of the frontline health workers**		
	2.1	How do you manage stress at work?
	2.2	What types of support does your workplace provide to help you manage stress?
**Specific aim 3: to understand challenges of stress management**		
	3.1	What challenges do you face while managing stress at work during the pandemic?

A total of 25 survey responses were collected. Since the number of individuals who were part of the mailing list is unknown, the response rate cannot be determined. Five response entries were dropped because the questions were inadequately answered (eg, one word or irrelevant response) or left blank (eg, by typing “N/A”). In other words, the completion rate was 80%. The selected responses were 20 (female, n=18) FHWs from 12 US states and the District of Columbia. In total, 18 participants were employed, 1 was a student/intern, and 1 was on high-risk leave at the time of the survey. The ages ranged from 24 to 56 years, while years of experience ranged from 1 to 28 years. According to their occupation, 12 participants were registered nurses (RN) or nurse practitioners (NP), 2 were physician assistants (PA), 4 were certified nurse assistants (CNA), and 2 were respiratory therapists (RT).

### Data Analysis

Two researchers used qualitative content analysis [27] to elicit user needs and design requirements from the interview transcripts and survey responses. The researchers first independently used initial coding and memoing to code keywords that represented stressors and coping strategies of FHWs by reading and re-reading the qualitative data independently. The researchers then met face-to-face to compare their codes and resolve conflicts through discussions. The final codes were first consolidated under categories and then categories were combined into themes presented below.

## Results

### Stressors

We identified four key challenges and concerns that FHWs believed were elevating stress levels during the peak of the COVID-19 pandemic. The most frequently occurring stressor has been described first, followed by the second most frequently occurring code, and so on. The fourth stressor is not exclusively related to the pandemic, but it uncovers an important source of stress among FHWs during normal situations.

#### Coping With Uncertainty

FHWs reported that coping with the uncertainty surrounding the pandemic was extremely stressful. Earlier during the pandemic, hospitals had not had time to revise their guidelines. FHWs did not know how to tackle the infection and how to manage patients. The absence of essential knowledge in response to patients’ needs was usual among the FHWs. One FHW remarked the following [28]:

Everyone had to struggle with the gap between what we believed were the proper procedures and what was possible during the crisis.

Many participants had to join specialties outside their areas of expertise to address the issue of shortage of FHWs and to serve the patients [29]. Adjusting to the new work environment was difficult for those who had been displaced from their normal work environments. Those who were working in their usual environments found it stressful to manage patient’s demands. One FHW reported the following to the Seattle Times [30]:

If patients have certain symptoms and questions ... we don’t have all the knowledge to answer the questions.

FHWs complained that as the pandemic progressed, they had to cope with frequent changes in rules and regulations. Adjusting to and staying abreast of all the updates in the heat of the pandemic was challenging for many FHWs.

Adjusting to changing rules and recommendations when you do not know what they are can become a huge stressor. (CNA1 at a surgery center).

FMWs must work in extremely uncertain and life-threatening circumstances. This stressor can be challenging to address in cases of novel pandemics when there are too many unknowns, and the knowledge is evolving. However, this theme shows that there is a paucity of efficient methods for disseminating necessary and evolving information to FHWs.

#### Fear of Contracting Infection

Generally speaking, to avoid the contagion, health workers utilize different types of personal protective equipment (PPE) including face masks, face shields, gloves, goggles, gowns, head covers, and shoe covers. Owing to the global shortage of PPE, many FHWs were working with low-grade and inadequate PPE; for example, PPE that leave necks and most of the face exposed. A nurse explained the following to the New York Times [31]:

We’ve been put on the front line not only without enough protection, but also sometimes with the stress of a very different work environment.

This resulted in many FHWs fearing contracting the infection while serving COVID-19–positive patients at the hospitals and health facilities [32].

The direct exposure to Covid+ patients exposes us to the risk of infection on a daily basis.

CNA1

Related to this, the FHWs were afraid of contracting the infection and then spreading it to family members including children and older adults at home. Many health care workers were taking extreme measures such as not going home and staying in tents and makeshift homes to protect their loved ones. The inability to go home and be with their families was an added stressor for the health care workers. Those who managed to go home were taking extra precautions such as stripping down and putting the hospital clothes in the laundry to ensure they were not exposing their loved ones to the risk of infection. It was challenging to stop feeling the guilt of possibly risking family members’ lives. An emergency department nurse spoke anonymously during an interview and reported the following [33]:

I live with my pregnant wife, son, and two dogs. My wife is also a nurse. We try to strip down as soon as possible when coming in the door, clothes straight to the laundry.

FHWs may need appropriate tools to monitor their health and provide greater insight into risk factors for reduced physical and mental health. Moreover, FHWs need to understand how to transact their feelings related to changed family dynamics during the pandemic.

#### Moral Injury

Several interviews described that as uninsured patients were dying, FHWs were asking who would pay for the uncovered health care expenses. Often, this implied that FHWs were making decisions about who should and should not get the ventilator. Whenever patients died in these situations, and many died without their loved ones by their sides, FHWs experienced guilt, compassion fatigue, and moral injury as they were unable to provide standard care and treatment to the patient.

There are many circumstances that can pose great psychological burden on FHWs and without a proper understanding of how to manage themselves, FHWs are likely to burn out owing to psychological burden.

#### Lack of Experience

The FHWs who were in the early stages of their career pointed out that the lack of experience was already a stressor for them. The pandemic created a work environment where finding answers to questions became even more challenging, causing stress levels of exacerbate.

Working in the ER can be stressful especially early on in my career. Each new experience can be stressful and scary because I don’t have a previous encounter with it. Sometimes it is hard to manage stress in some situations because I lack experience, so I feel like that makes the situation worse.

Emergency Department RN3

FHWs who are in the early stages of their career may need additional support during public health emergencies to cope with their lack of experience along with specific emergency-related challenges.

### Stress Management Strategies

We found four main stress management strategies that were used by participants: self-care, teamwork, counseling services, and internal motivation. We describe these strategies below in accordance with the frequency (highest to lowest) with which these strategies were reported.

#### Quick Coping

Based on their preferences and time allowances, participants used various quick coping strategies to manage stress. “*Taking breaks*” (n=12) and “*walking away*” (n=12) from stressful situations were the most frequently used strategies.

I try to find moments to step away. Take deep breath and try to remember I can only do one patient at a time.

Emergency Room RN4

While, for many participants, simply stepping away from a stressful situation was enough to refocus and destress, others engaged in health-promoting activities such as yoga, meditation, listening to music, and watching funny videos on YouTube. Six participants also reported using mobile apps such as Calm and HeadSpace to distract themselves from stressful events [34].

Maintaining daily routines such as regular exercise, clean sleep schedule and periodic meditation sessions.

Other participants mentioned strategies such as “*calling a loved one*,” “*stress eating*,” and “*attending social events*” to manage stress levels at work, suggesting that there are many coping methods that are simple and readily available to FHWs.

#### Teamwork

There was a consensus among the surveyed FHWs that sharing their frustrations and experiences with peers was important for them as it kept them grounded and made it easier for them to deal with the challenges of the pandemic. Everyone believed in supporting one other and creating a safe space to help each other get through the tough times.

Laughing and venting out with co-workers is my way to manage stress at work.

RN5

Moreover, participants relied on teamwork and support of their colleagues at work whenever they wanted to find answers to their work-related questions or manage stressful situations at work.

When I am at work, I connect with team members, ask for help when I feel overloaded. I try to help others the same way.

RN6

I have coworkers that are open to listen and help in any situation.

Emergency Room RN2

When peers share their challenges and frustrations about common issues with each other, they acquire the strength to overcome stress. Gaining support and attention of their peers is important for FHWs. They might need more effective ways to share problems and connect with each other.

#### Counseling Support

Many FHWs reported that they were joining weekly virtual healing circles to learn effective strategies to cope with different stressors. One article highlighted a hotline service that was specifically created to support FHWs with distress and mental fatigue. A counselor on a hotline stated that the most common calls from FHWs were related to stress, exhaustion, and worries about families, particularly having to stay away from family members for safety. This theme implies that FHWs actively strive to find resources to handle their problems before, during, and after the stressful situations.

#### Building Resilience

FHWs mentioned that they focus on building resilience when they feel challenged or overwhelmed at work. In fact, having internal motivation and drive was an expectation of their work environment, which every FHW considered seriously.

When things get tough, you power through.

Rapid Response RN7

FHWs actively engaged in educating themselves about the unique pressures, fears, and demands about the COVID-19 pandemic and even attended training sessions to cope, persevere, and survive challenges of each day. One FHW reported the following [34]:

Find peace in preparation and educating oneself about how to deal with stressors. Have a mantra – something that strengthens and brings calm at the same time.

FHWs might place particular stress on developing their internal motivations to solve their problems. They appreciate having access to training, which can help them develop their internal motivation.

### Challenges of Managing Stress

Two main challenges were mentioned by the FHWs and have been described in accordance with their frequency (highest to lowest) of occurrence in the collected data.

#### Time Constraints

During the peak of the pandemic, FHWs had to contend with the high patient load and short staffing issues. As a result, FHWs found themselves working without breaks and without clear end in sight. Many FHWs reported that they had been unable to find time to focus on their own physical and mental health.

Not having enough time to mentally care for oneself while providing 110% care for a patient (or 2 in the ICU) during a 12-14hr shift.

Intensive Care Unit RN1

Many FHWs reported that their routine had been disrupted and they could not engage in routine daily activities at regular times such as meal breaks, sleep, and physical exercises.

The biggest challenge has been balancing immediate patient needs with immediate personal needs, including basic things like taking time to eat, drink and use the bathroom.

Emergency Room RN2

Several FHWs also wanted to engage in productive activities such as attending lectures or seminars on positive thinking, effective time management, etc; however, owing to the heavy workload and back-to-back shifts, they were unable to do so.

Too much work and high activity patients do not leave enough time to go to lectures or events.

NP1

This theme reminds us of the importance of designing interventions that fit effectively into the work life of FHWs. FHWs may understand what their needs are, but time is a scarce commodity. Without time-efficient methods, seeking and utilizing necessary resources to address their needs is bound to remain a challenge for FHWs.

#### Management’s Limited Capacity

Participants pointed out that during crises, management is also subject to stress and health system inadequacies, which impacts their ability to make appropriate adjustments and changes. In some cases, this resulted in lack of enough resources and support that were desired by FHWs to cope with stress due to the pandemic.

Management is unwilling to provide ways to alleviate stress.

PA1

I was told at one point to provide my own masks. I intake patients and start their IVs and somehow my employer felt they shouldn’t be accountable for providing me with a surgical mask daily.

CNA2

On the other hand, some FHWs pointed out that although the employers provided many mental health benefits, these resources were not well-advertised. Hence, many FHWs who required mental health support felt that they needed more commitment from their employers in terms of addressing their mental health needs.

This theme suggests that FHWs want their authorities to provide guidance during the times of stress; however, they might be hesitant to ask for help and resources directly from their management. Moreover, management might not know what kind of resources are needed by their employees.

## Discussion

### Principal Findings

We identified 4 major stressors, 4 stress management strategies, and 2 stress management challenges among FHWs that are specifically related to pandemics as well as general situations. Uncertainty and an unstable work environment owing to frequent changes in work protocols owing to the pandemic and a fear of contracting the infection caused considerable stress among FHWs. Moral injury associated with seeing patients die from lack of care and inexperience with handling other circumstances that occur in health care settings were other sources of stress. FHWs used simple coping strategies such as walking away from stressful situations, entertaining oneself, and exercising to mitigate the impact of stress at work. Peer support and counseling services were other popular methods to learn about strategies to minimize the burden of stress. Building resilience and driving oneself forward using internal motivation were also meaningful ways of overcoming stressful situations. However, time constraints, management’s limited initiative taking, and lack of resources prevented FHWs from engaging in activities that could lessen the impact of stress.

### Practical Implications

Our findings show that FHWs experience professional, personal, and social types of stressors in their workplaces during the pandemic. While they can manage certain types of stressors, it is difficult for them to manage other types. Specifically, it is challenging for them to cope with uncertainty, which was the most frequently recounted stressor, and was the subtext in all the other stressors. That is, FHW felt uncertain about their personal health situation, uncertain about their role in providing full care to underprivileged infected patients, and uncertain about handling novel and challenging scenarios that the pandemic created in their work environments. Understanding how other health care professionals manage uncertainty and the resulting stress in their work practices can provide directions for designing interventions for FHWs. For example, research shows that genetic counselors (GCs) routinely encounter uncertainty in their practice, which forces them to engage in a variety of strategies to manage it. Specifically, GCs resort to seeking information, identifying social support, and normalizing uncertainty to cope with uncertainty [35]. Basher’s Theory of Uncertainty Management [36] explains that individuals can have negative, positive, or neutral responses to uncertainty and they manage their uncertainty by adopting the aforementioned strategies; that is, knowledge, social support, and normalization. FHWs can be supported with similar strategies to help them manage uncertainty and consequently stress during the pandemic. Below we discuss how mobile apps can be used to support such strategies.

FHWs in our study could not overcome their uncertainty by seeking information because of it was evolving and changing in the heat of the pandemic. The existing COVID-19 apps [17-20] reviewed in the beginning of this paper incorporate a wide array of knowledge resources for health workers but there is no research about the effectiveness of these resources. For example, how accessible are they for handling specific situations and scenarios? The health workers in our study indicated that lack of time prevented them from taking advantage of the resources around them. Therefore, while mobile apps can reach the health workers with appropriate resources “where they are,” strategies are needed to ensure these resources are actionable and effectively utilized.

Mobile apps can increase opportunities for FHWs to seek social support by interacting with their peers to fulfill their emotional and professional needs in times of crisis. Earlier research shows that FHWs appreciate access to an in-app peer support community [37]. Cheng et al [38] had proposed a peer-to-peer psychological support and crisis management infrastructure based on popular social media apps such as WeChat. We extend this proposal by suggesting that FHWs should be able to seek support and answers to their queries from experts within and beyond their institutional boundaries. Care must be taken to ensure that these peer support tools are designed to prevent negative social pressure and other potential risks of digital interactions [37]. Moreover, peer support requires peer time, posing burden on other peers. A possibility is automating peer support by using; for example, artificially intelligent tools such a conversational agent. Institutions can also investigate developing institution-specific conversational agents to help FHWs with personalized searches.

Building resilience and adopting quick coping strategies may be seen as FHWs’ effort to normalize uncertainty and deal with psychological issues such as guilt and moral injury. However, our findings also indicate that FHWs reach out to counseling services for support suggesting that certain psychological needs of FHWs are not readily met. Mobile apps can be used to deliver psychological interventions and have shown effectiveness in the past. Ly et al [39] showed that Acceptance Commitment Therapy delivered via smartphones can reduce perceived stress, increase general health, and promote psychological flexibility in business managers [40]. However, none of the COVID-19 apps reported earlier in this paper used any such theoretically driven stress management strategy. Further research should focus on understanding other psychological needs of the FHWs and developing appropriate interventions to address those needs.

### Theoretical Implications

Our results corroborate the existing theoretical frameworks on stress mechanisms and management. The transactional framework conceptualizes stress as an internal representation of a problematic transaction between the person and their work environment [41]. On the one hand, intervention for stressors can reside in the workplace; on the other, interventions or coping are at the individual level. Given that our findings recommend an individual level strategy, our study supports the constructs of transactional theory to mitigate frontline challenges.

Beyond corroborating the existing theoretical frameworks on stress mechanisms and management, this study advances research and theories on stress management in 2 ways. First, the extant research focuses on stress management from a general perspective, we have identified stressors, and coping strategies, specifically in FHWs. Second, we have applied the Theory of Uncertainty Management to suggest strategies to reduce stress in FHWs. In this sense, we have expanded the definition of stress from being a problematic transaction between an individual and their work environment to also include the cognitive state that develops due to ambiguity and unpredictability. This understanding has practical implications as previously discussed.

### Limitations and Future Work

The work presented here has several limitations that can be addressed in future studies. First, this study does not consider organizational perspective and challenges they face in implementing effective stress management strategies for their workers during the pandemic. Previous work has shown that there are often misalignments between the mental health needs of the employees and what employers’ can financially or legally provide [42]. Therefore, a future study can explore health organization’s concerns to further refine the presented design suggestions. Second, an important consideration in future research is how geographic location may affect the stressors, strategies, or user needs amid the pandemic. Research has shown that there are differences in health workers’ qualifications in urban versus rural areas [43]. These differentials directly impact the level of knowledge and willingness to work during crisis situations [44]. Hence, there might be different challenges and strategies that have been overlooked in this study. Third, there is a possibility that the selection of the article content was influenced by our biases (none of the researchers was a healthcare professional). Therefore, future research in this area should confirm the findings with health care experts or other key informants. However, the logistics of such an endeavor might be challenging to implement in practice. Finally, a quantitative measure-based study may provide generalizable insights that cannot be achieved via qualitative studies. This would require conducting a large-scale survey with FHWs from varied settings.

### Conclusions

FHWs encounter stressful situations in their workplace and need effective strategies to cope with these stressors. The goal of this study was to provide suggestions for designing mobile apps to address workplace stressors of FHWs based on the understanding of their needs from FHWs’ own perspectives and the Transactional Framework of Stress. We identified four major stressors including coping with uncertainty, fear of infection, moral injury, and lack of experience in managing various challenges. The uncovered coping strategies comprised quick coping, peer support, counseling services and building resilience. Time constraints and management’s limited capabilities are some challenges that FHWs encounter while managing stress. The findings corroborate the existing theoretical frameworks on stress mechanisms and advance the understanding of stress management from the FHWs’ perspective. We extend the definition of stress to include uncertainty and discuss how principles of uncertainty management can provide directions for designing stress management apps for FHWs. Specifically, mHealth apps can be designed to help FHWs seek peer support and information. They can also be used to deliver specific psychological interventions for FHWs. Given that the pandemic is ongoing and crisis at work will continue, FHWs remain a vulnerable population in need of attention. Hence, our efforts have implications for advancing the ongoing efforts aimed at improving FHWs’ mental well-being worldwide.

## Abbreviations

CNA: certified nurse assistant
FHW: frontline health worker
GC: genetic counselor
NP: nurse practitioner
PA: physician assistant
PPE: personal protective equipment
RN: registered nurse
RT: respiratory therapist
