# Correction: Toward Designs of Workplace Stress Management Mobile Apps for Frontline Health Workers During the COVID-19 Pandemic and Beyond: Mixed Methods Qualitative Study

**DOI:** 10.2196/36769

**Published:** 2022-01-25

**Authors:** Beenish Moalla Chaudhry, Ashraful Islam, Monica Matthieu

**Affiliations:** 1 University of Louisiana at Lafayette School of Computing and Informatics Lafayette, LA United States; 2 Saint Louis University College for Public Health and Social Justice, School of Social Work Saint Louis, MO United States

In “Toward Designs of Workplace Stress Management Mobile Apps for Frontline Health Workers During the COVID-19 Pandemic and Beyond: Mixed Methods Qualitative Study” (JMIR Form Res 2022;6(1):e30640) the authors noted one error.

In the originally published manuscript, the title was incorrectly set as:

Toward Designs of Workplace Stress Management Mobile Apps for Frontline Health Workers During the COVID-19 Pandemic and Beyond: Design Implications for a Mixed Methods Qualitative Study

The title has been corrected to:

Toward Designs of Workplace Stress Management Mobile Apps for Frontline Health Workers During the COVID-19 Pandemic and Beyond: Mixed Methods Qualitative Study

The correction will appear in the online version of the paper on the JMIR Publications website on January 25, 2022, together with the publication of this correction notice. Because this was made after submission to PubMed, PubMed Central, and other full-text repositories, the corrected article has also been resubmitted to those repositories.

